# Pro-life role for c-Jun N-terminal kinase and p38 mitogen-activated protein kinase at rostral ventrolateral medulla in experimental brain stem death

**DOI:** 10.1186/1423-0127-19-96

**Published:** 2012-11-17

**Authors:** Alice YW Chang

**Affiliations:** 1 Center for Translational Research in Biomedical Sciences, Kaohsiung Chang Gung Memorial Hospital, Kaohsiung, 83301, Taiwan, Republic of China

## Abstract

**Background:**

Based on an experimental brain stem death model, we demonstrated previously that activation of the mitogen-activated protein kinase kinase 1/2 (MEK1/2)/extracellular signal-regulated kinase 1/2 (ERK1/2)/ mitogen-activated protein kinase signal-interacting kinase 1/2 (MNK1/2) cascade plays a pro-life role in the rostral ventrolateral medulla (RVLM), the origin of a life-and-death signal detected from systemic arterial pressure, which sequentially increases (pro-life) and decreases (pro-death) to reflect progressive dysfunction of central cardiovascular regulation during the advancement towards brain stem death in critically ill patients. The present study assessed the hypothesis that, in addition to ERK1/2, c-Jun NH2-terminal kinase (JNK) and p38 mitogen-activated protein kinase (p38MAPK), the other two mammalian members of MAPKs that are originally identified as stress-activated protein kinases, are activated specifically by MAPK kinase 4 (MAP2K4) or MAP2K6 and play a pro-life role in RVLM during experimental brain stem death. We further delineated the participation of phosphorylating activating transcriptional factor-2 (ATF-2) and c-Jun, the classical transcription factor activated by JNK or p38MAPK, in this process.

**Results:**

An experimental model of brain stem death that employed microinjection of the organophosphate insecticide mevinphos (Mev; 10 nmol) bilaterally into RVLM of Sprague–Dawley rats was used, alongside cardiovascular, pharmacological and biochemical evaluations. Results from ELISA showed that whereas the total JNK, p38MAPK, MAP2K4 and MAP2K6 were not affected, augmented phosphorylation of JNK at Thr183 and Tyr185 and p38MAPK at Thr180 and Tyr182, accompanied by phosphorylation of their upstream activators MAP2K4 at Ser257 and Thr261 and MAP2K6 at Ser207 and Thr211 in RVLM occurred preferentially during the pro-life phase of experimental brain stem death. Moreover, the activity of transcription factors ATF-2 at Thr71 and c-Jun at Ser73, rather than Elk-1 at Ser383 in RVLM were also augmented during the pro-life phase. Furthermore, pretreatment by microinjection into the bilateral RVLM of specific JNK inhibitors, JNK inhibitor I (100 pmol) or SP600125 (5 pmol), or specific p38MAPK inhibitors, p38MAPK inhibitor III (500 pmol) or SB203580 (2 nmol), exacerbated the depressor effect and blunted the augmented life-and-death signal exhibited during the pro-life phase. On the other hand, pretreatment with the negative control for JNK or p38MAPK inhibitor, JNK inhibitor I negative control (100 pmol) or SB202474 (2 nmol), was ineffective in the vehicle-controls and Mev-treatment groups.

**Conclusions:**

Our results demonstrated that activation of JNK or p38MAPK in RVLM by their upstream activators MAP2K4 or MAP2K6 plays a preferential pro-life role by sustaining the central cardiovascular regulatory machinery during experimental brain stem death *via* phosphorylation and activation of nuclear transcription factor ATF-2 or c-Jun.

## Background

Whereas brain stem death is the legal definition of death in the United States of American [1], United Kingdom [2], European [3], Taiwan and many other countries [1,4], the detailed cellular and molecular mechanisms underlying this phenomenon of prime medical importance are only begun to emerge. Since asystole invariably occurs within hours or days after the diagnosis of brain stem death [5], it is strongly suggested that permanent impairment of the brain stem cardiovascular regulatory machinery precedes death [6]. Further understanding of the mechanisms of this aspect of cardiovascular regulatory dysfunction should therefore enrich the dearth of information currently available on brain stem death.

Mitogen-activated protein kinases (MAPKs) are serine/threonine-specific protein kinases that regulate proliferation, gene expression, differentiation, cell survival and apoptosis [7]. Three most widely characterized MAPK subfamilies are extracellular signal-regulated kinase 1/2 (ERK1/2), c-Jun NH2-terminal kinase (JNK) and p38MAPK [8]. Activation of MAPKs requires phosphorylation of its regulatory loop by upstream activators. Thus, each of these subfamilies is composed of MAPK kinase kinase (MAP3K) that, on activation, phosphorylates a MAPK kinase (MAP2K), then a MAPK. The phosphorylated MAPK interacts with its cellular substrates, which translocate to the nucleus to modulate transcription factors that results in a diverse range of biological responses.

Based on a clinically relevant animal model of brain stem death [6,9] in conjunction with toxicity elicited by the organophosphate insecticide mevinphos (3-(dimethoxyphosphinyloxyl)-2-butenoic acid methyl ester (Mev), a US Environmental Protection Agency Toxicity Category I pesticide, we demonstrated previously that the rostral ventrolateral medulla (RVLM) is a suitable neural substrate for mechanistic evaluation of this fatal phenomenon [6], because it is the origin of a life-and-death signal [10] that reflects failure of the central cardiovascular regulatory machinery during brain stem death [11-13] and is a brain stem site *via* which Mev acts to elicit cardiovascular toxicity [9]. Of interest is that the waxing and waning of the life-and-death signal, which mirrors the fluctuation of neuronal functionality in RVLM, presents itself as the low-frequency (LF) component in the systemic arterial pressure (SAP) spectrum of comatose patients [11-13]. More importantly, the distinct phases of augmentation followed by reduction of the LF power exhibited during Mev intoxication [14-17] can be designated the pro-life and pro-death phase of central cardiovascular regulation in this model of brain stem death [6]. Based on this model, our laboratory has previously demonstrated that activation of MAPK kinase 1/2 (MEK1/2) in RVLM, followed by ERK1/2 and MAPK signal-interacting kinase 1/2 (MNK1/2) activation, is responsible for the pro-life phase by sustaining the central cardiovascular regulatory machinery during brain stem death [18,19].

Of the three MAPKs characterized in mammals, JNK and p38MAPK are originally identified as a stress-activated protein kinase (SAPK) that primarily mediates inflammatory response [20,21] and promotes cell death [22-24]. However, recent studies further suggest that JNK and p38MAPK may also participate in cell survival [25-27], proliferation [28] or pressor response [29]. With particular relevance to the present study, simultaneous inhibition of JNK and p38MAPK increases cell death in the heart of rats induced by ischemia/reperfusion injury [30]. Moreover, activation of p38MAPK signaling pathway in RVLM underlies the pressor response to angiotensin II (Ang II) in rats [29].

As death represents the end of existence for an individual, we proposed previously [6] that multiple pro-life and pro-death programs must be activated in RVLM during the progression toward brain stem death. Furthermore, we previously demonstrated that ERK1/2 in RVLM plays a pro-life role in experimental brain stem death [18,19]. In our continual search for the cellular and molecular underpinning of brain stem death, the next logical direction is to evaluate the contribution of the other two family members of MAPKs, JNK or p38MAPK in RVLM to this fatal phenomenon.

Based on our Mev intoxication model [6], the present study evaluated the hypothesis that JNK and p38MAPK in RVLM play a pro-life role during brain stem death. We further delineated the upstream participation of MAPK kinase 4 (MAP2K4) and MAPK kinase 6 (MAP2K6) and downstream participation of transcription factors activating transcriptional factor-2 (ATF-2) and c-Jun, the nuclear substrates of JNK or p38MAPK [31] in this process. Our results demonstrated that activation of JNK and p38MAPK in RVLM plays a preferential pro-life role by sustaining central cardiovascular regulatory functions during brain stem death. We further found that the signaling cascade for the pro-life process includes upstream phosphorylation of MAP2K4 or MAP2K6, and downstream activation of transcription factors ATF-2 or c-Jun.

## Methods

Adult male Sprague–Dawley rats (275–350 g, n = 129) purchased from the Experimental Animal Center of the National Science Council, Taiwan, Republic of China were used. They were housed in our Association for Assessment and Accreditation of Laboratory Animal Care (AAALAC)-International accredited Center for Laboratory Animals. All animal care and experimental procedures carried out in this study have been approved by the Institutional Animal Care and Use Committee of the Kaohsiung Chang Gung Memorial Hospital, and were in compliance with the guidelines of this Committee. Animals were housed in groups of two to three in individually ventilated cages, in a temperature-controlled room (22 ± 2°C) with 12 h light/12 h dark cycles (lights on at 07:00 h), with free access to rat chow and water. All efforts were made to minimize animal suffering and to reduce the number of animal used.

### General preparation

After application of an induction dose of pentobarbital sodium (50 mg/kg, i.p.), preparatory surgery, including cannulation of a femoral artery and a femoral vein, together with tracheal intubation, was carried out. During the recording session, which routinely commenced 60 min after the administration of pentobarbital sodium, anesthesia was maintained by intravenous infusion of propofol (Zeneca, Macclesfield, UK) at 20–25 mg/kg/h. We have demonstrated previously [32] that this scheme provided satisfactory anesthetic maintenance while preserving the capacity of central cardiovascular regulation. Rats were allowed to breathe spontaneously with room air and body temperature of rats was maintained at 37°C with a heating pad.

### Animal model of brain stem death

The Mev intoxication model of brain stem death [6] that we established previously was used. Since Mev induces comparable cardiovascular responses on given systemically or directly to RVLM [9], we routinely microinjected Mev bilaterally into RVLM to elicit site-specific effects [9,14-17]. SAP signals recorded from the femoral artery were simultaneously subject to on-line power spectral analysis (SPA10a; Notocord, Croissy-Sur-Seine, France) [9,14-17,33]. We were particularly interested in the LF component (0.25–0.8 Hz) in the SAP spectrum because its power density mirrors the prevalence of baroreflex-mediated sympathetic neurogenic vasomotor discharges that emanate from this brain stem site [33]. More importantly, our laboratory demonstrated previously [14-17] that the power density of this spectral signal exhibits biphasic changes that reflect the pro-life and pro-death phases seen during the progression towards brain stem death in patients who succumbed to organophosphate poisoning [13]. Heart rate (HR) was derived instantaneously from SAP signals. Temporal changes in the power density of the LF component, pulsatile SAP, mean SAP (MSAP) and HR were routinely followed for 180 min after Mev administration in an on-line and real-time manner.

### Microinjection of test agents

Microinjection bilaterally of test agents into RVLM, each at a volume of 50 nl, was carried out stereotaxically and sequentially [9,14-17]*via* a glass micropipette connected to a 0.5-μl Hamilton (Reno, NV, USA) microsyringe. The coordinates used were: 4.5–5 mm posterior to lambda, 1.8–2.1 mm lateral to midline, and 8.1–8.4 mm below the dorsal surface of cerebellum. These coordinates were selected to cover the ventrolateral medulla at which functionally identified sympathetic premotor neurons reside [34]. Test agents used included Mev (kindly provided by Huikwang Corporation, Tainan, Taiwan), two specific JNK inhibitors, JNK inhibitor I (Calbiochem, San Diego, CA, USA) [35] and JNK inhibitor II (SP600125, Calbiochem) [36]; two specific p38MAPK inhibitors, p38 MAPK inhibitor III (Calbiochem) [37] and SB203580 (Calbiochem) [38]; and negative controls, JNK inhibitor I negative control (Calbiochem) [35] or SB202474 (Calbiochem) [38]. All test agents used for pretreatment were given 30 min before the administration of Mev. The doses were adopted from previous reports [35-38] that used those test agents for the same purpose as in this study. Application of the same amount of artificial cerebrospinal fluid (aCSF) controlled for possible volume or solvent effect. The composition of aCSF was (mmol/L): NaCl 117, NaHCO_3_ 25, glucose 11, KCl 4.7, CaCl_2_ 2.5, MgCl_2_ 1.2 and NaH_2_PO_4_ 1.2. To avoid the confounding effects of drug interactions, each animal was subject routinely to only one pharmacological treatment scheme.

### Collection of tissue samples from ventrolateral medulla

As in previous studies [14-17], we routinely collected tissue samples for subsequent biochemical evaluations during the peak of the pro-life phase and pro-death phase (Mev group), or 30 or 180 min after microinjection of aCSF into RVLM (vehicle control group). Animals were killed with an overdose of pentobarbital sodium and tissues from both sides of the ventrolateral medulla, at the level of RVLM (0.5–1.5 mm rostral to the obex), were collected by micropunches made with a 1 mm (i.d.) stainless-steel bore to cover the anatomical boundaries of RVLM. Medullary tissues collected from anesthetized animals without any treatment served as the sham-controls. The concentration of total proteins extracted from tissue samples was determined by the BCA protein assay (Pierce, Rockford, IL, USA).

### ELISA for protein level of JNK, p38MAPK, MAP2K4, MAP2K6 or their phosphorylated forms

Cell lysate from ventrolateral medulla was subject to a commercial kit for enzyme-linked immunosorbent assay (ELISA) according to the manufacturer’s protocol to detect the levels of JNK1/2/3 (eBioscience, San Diego, CA, USA), phosphorylated JNK1/2/3 at Thr183/Tyr185 (eBioscience), p38MAPK (eBioscience), phosphorylated p38MAPK at Thr180/Tyr182 (eBioscience), MAP2K4 (Antibodies-online, Atlanta, GA, USA), phosphorylated MAP2K4 at Ser257/Thr261 (TGR BioSciences, Thebarton, Australia), MAP2K6 (MyBioSource, San Diego, CA, USA) or phosphorylated MAP2K6 at Ser207/Thr211 (R and D Systems, Minneapolis, MN, USA). The final absorbance of reaction solution at 450 nm was determined by spectrophotometry using an ELISA microtiter plate reader (Anthros Labtec, Salzburg, Austria), and was expressed as fold changes against baseline-controls.

### Nuclear extract from ventrolateral medulla

In some experiments, proteins from the nuclear fraction of the medullary samples were extracted using a commercial kit (Active Motif, Carlsbad, CA, USA). The concentration of protein in the nuclear extracts was again estimated by the BCA Protein Assay (Pierce).

### ELISA for activity of transcription factors ATF-2, c-Jun or Elk-1

Nuclear extract from ventrolateral medulla was subject to a sensitive and specific commercial kit (Active Motif) for ELISA according to the manufacturer’s protocol to detect the levels of phosphorylated c-Jun at Ser73, phosphorylated E twenty-six-like transcription factor 1 (Elk-1) at Ser383 or phosphorylated ATF-2 at Thr71. The final absorbance of the reaction solution at 450 nm was determined by spectrophotometry using an ELISA microtiter plate reader (Anthros Labtec), and expressed as fold changes against baseline-controls.

### Histology

In some animals that were not used for biochemical analysis, the brain stem was removed at the end of the physiological experiment and fixed in 30% sucrose in 10% formaldehyde-saline solution for at least 72 h. Frozen 25-μm sections of the medulla oblongata stained with neural red were used for histological verification of the microinjection sites.

### Statistical analysis

All values are expressed as mean ± SEM. The averaged value of MSAP or HR calculated every 20 min after the administration of test agents or aCSF, the sum total of power density for the LF component in the SAP spectrum over 20 min, or the level or activity of protein or transcriptional factor in RVLM during each phase of experimental brain stem death, were used for statistical analysis. One-way or two-way ANOVA with repeated measures was used, as appropriate, to assess group means. This was followed by the Scheffé multiple-range test for *post hoc* assessment of individual means. *P <*0.05 was considered to be statistically significant.

## Results

### Mev intoxication model of brain stem death

We demonstrated previously that co-microinjection bilaterally of Mev (10 nmol) and aCSF into RVLM elicited a progressive depressor effect that became significant 100 min after application, accompanied by indiscernible alterations in HR. Concurrent changes in the power density of the LF component of SAP signals revealed two distinct phases [14-17]. The pro-life Phase I (MI) entailed a significantly augmented LF power that endured 80–100 min to reflect sustained brain stem cardiovascular regulatory functions. The pro-death Phase II (MII), which lasted the remainder of our 180-min observation period, exhibited further and significant reduction in the power density of this spectral component to below baseline, which signifies failure of central cardiovascular regulation that precedes brain stem death [6].

### Preferential activation of JNK in RVLM during the pro-life phase

**Figure 1:**
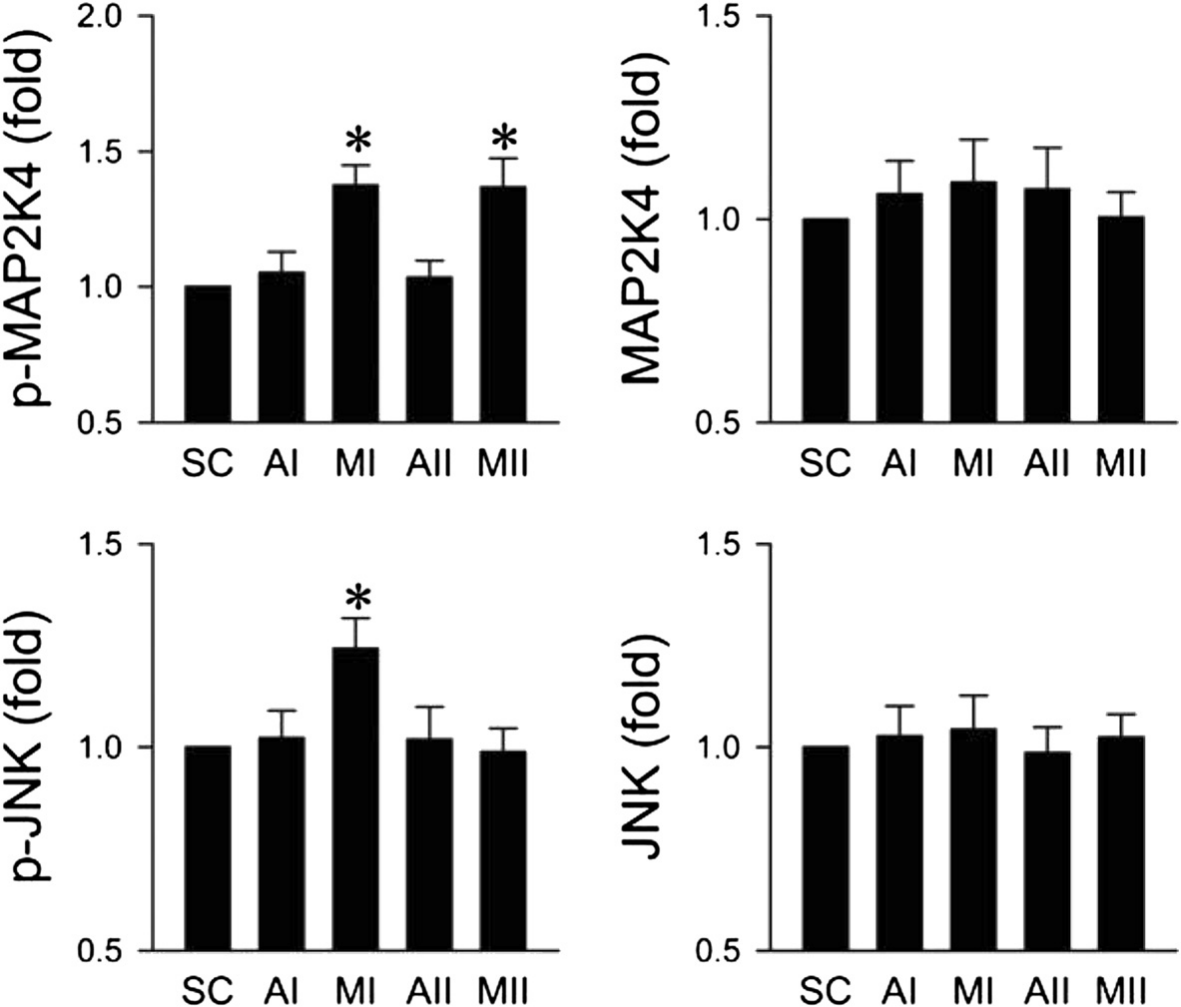
**Activation of JNK and MAP2K4 in RVLM during the pro-life phase of experimental brain stem death.** Changes in levels of total or phosphorylated JNK at Thr183 and Tyr185 and changes in levels of total or phosphorylated MAP2K4 at Ser257/Thr261 in folds relative to sham-control (SC), detected in ventrolateral medulla during the pro-life Phase I (MI) or pro-death Phase II (MII) during experimental brain stem death or during comparable time points after treatment with aCSF (AI or AII). Values are presented as mean ± SEM of triplicate analyses on tissue samples pooled from 5-7 animals in each experimental group. *P < 0.05 versus corresponding aCSF group in the post hoc Scheffé multiple-range analysis.

We first evaluated the fundamental premise that JNK in RVLM is activated during experimental brain stem death. Quantification by ELISA revealed that total JNK and its upstream activator MAP2K4 in ventrolateral medulla were not affected by microinjection of Mev into the bilateral RVLM (Figure 1). Interestingly, phosphorylated JNK (p-JNK) at Thr183 and Tyr185 in RVLM was significantly and preferentially augmented (Figure 1) during the pro-life phase of experimental brain stem death, which returned to baseline during the pro-death phase. However, phosphorylated MAP2K4 (p-MAP2K4) at Ser257/Thr261 was significantly increased (Figure 1) during both the pro-life and pro-death phases. The levels of JNK, MAP2K4 and phosphorylated JNK or MAP2K4 in ventrolateral medulla of vehicle groups 30 min (AI) or 180 min (AII) after aCSF application were comparable to sham-controls.

### Preferential activation of p38MAPK in RVLM during the pro-life phase

**Figure 2:**
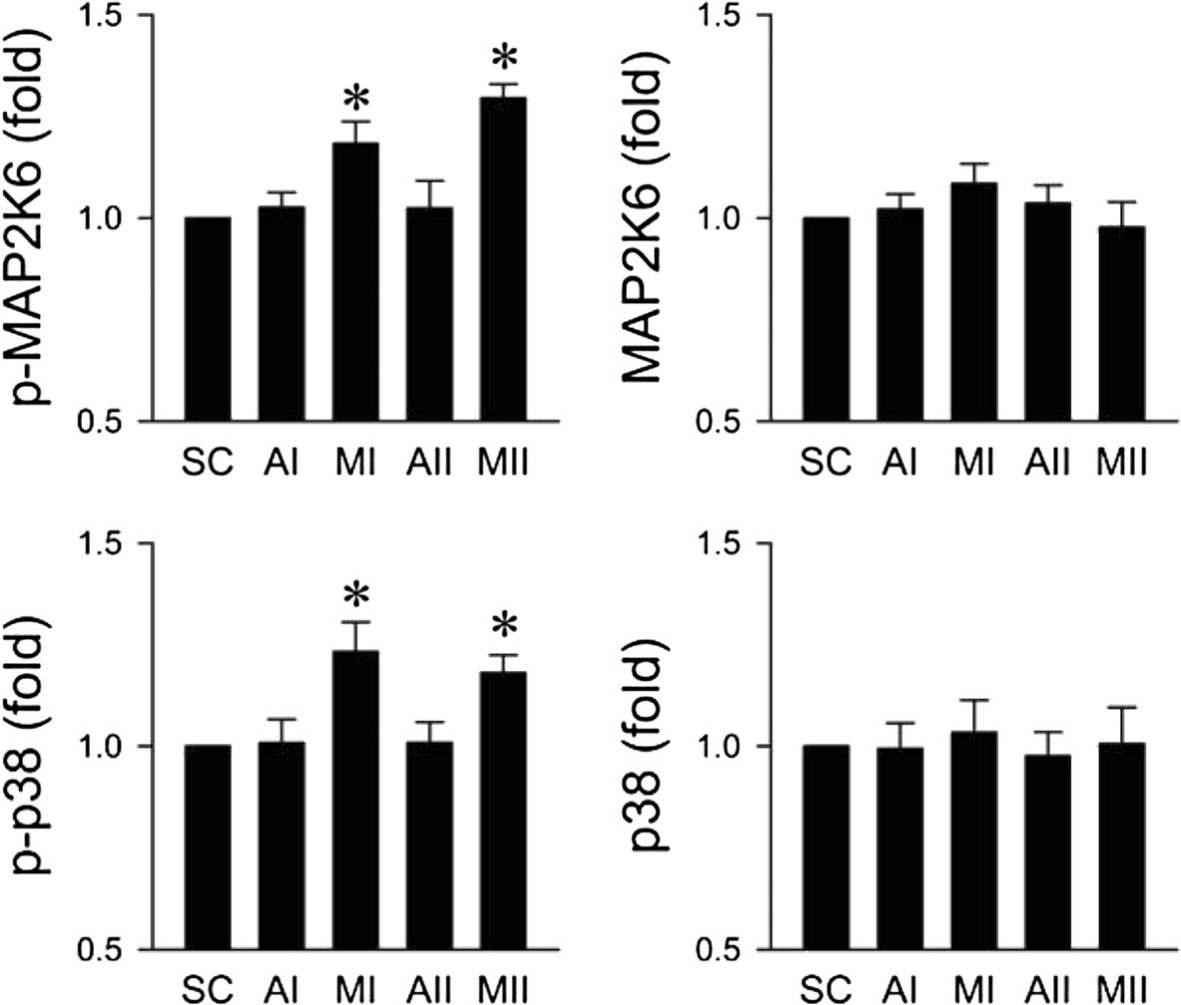
**Activation of p38MAPK and MAP2K6 in RVLM during the pro-life phase of experimental brain stem death.** Changes in levels of total or phosphorylated p38MAPK at Thr180 and Tyr182 and changes in levels of total or phosphorylated MAP2K6 at Ser207/Thr211 in folds relative to sham-control (SC), detected in ventrolateral medulla during the pro-life (MI) or pro-death (MII) phase of experimental brain stem death or during comparable time points in aCSF-controls (AI or AII). Values are presented as mean ± SEM of triplicate analyses on tissue samples pooled from 5-7 animals in each experimental group. *P < 0.05 versus corresponding aCSF group in the post hoc Scheffé multiple-range analysis.

We further evaluated whether p38MAPK in RVLM is also activated during experimental brain stem death. Quantification by ELISA again revealed that total p38MAPK and its upstream activator MAP2K6 in ventrolateral medulla were not affected by microinjection of Mev into the bilateral RVLM (Figure 2). Furthermore, both phosphorylated p38MAPK (p-p38MAPK) at Thr180/Tyr182 and phosphorylated MAP2K6 at Ser207/Thr211 in RVLM were significantly augmented (Figure 2) during both pro-life and pro-death phase. The levels of p38MAPK, MAP2K6 and phosphorylated p38MAPK or MAP2K6 in ventrolateral medulla of vehicle groups after aCSF application were comparable to sham-controls.

### Preferential activation of transcription factors c-Jun, ATF-2, rather than Elk-1 in RVLM during the pro-life phase

**Figure 3:**
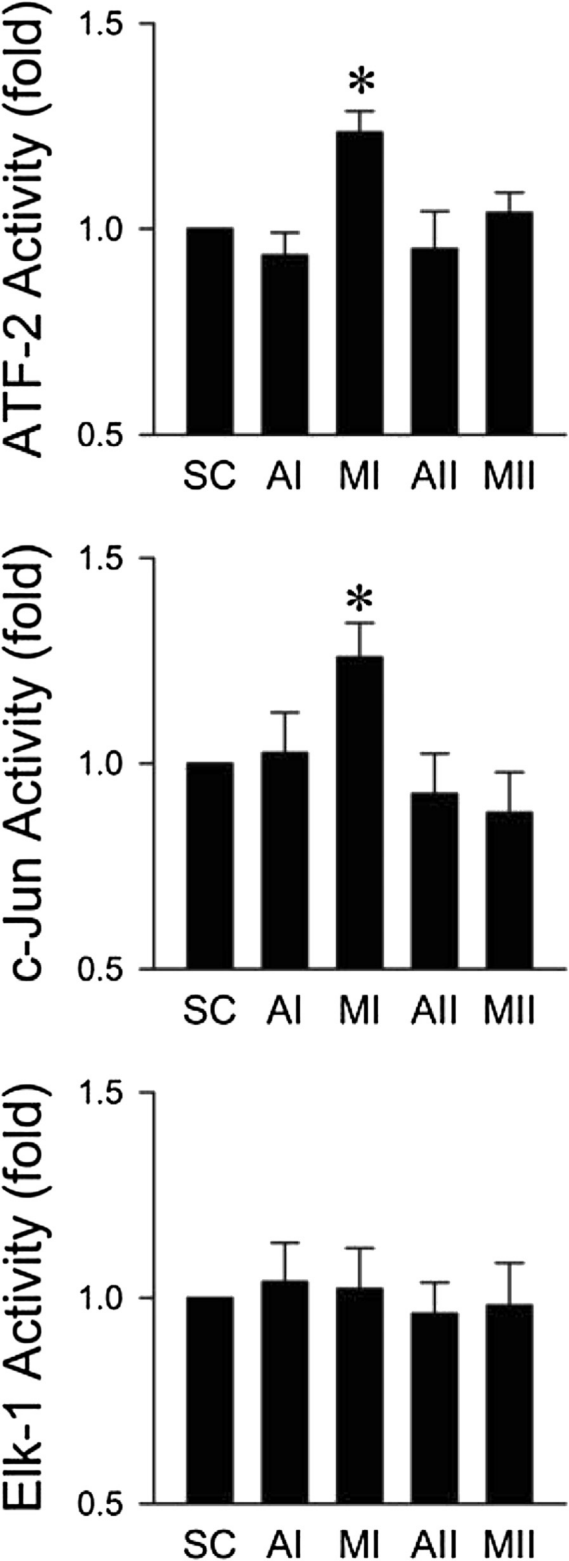
**Activation of transcription factor ATF-2, c-Jun, rather than Elk-1 in RVLM during the pro-life phase of experimental brain stem death.** Changes in the activity of ATF-2, c-Jun or Elk-1 represented by phosphorylation respectively at Thr71, Ser73 or Ser383, in folds relative to sham-control (SC), detected in ventrolateral medulla during the pro-life (MI) or pro-death (MII) phase of experimental brain stem death or during comparable time points in aCSF-controls (AI or AII). Values are presented as mean ± SEM of triplicate analyses on tissue samples pooled from 5-7 animals in each experimental group. *P < 0.05 versus corresponding aCSF group in the post hoc Scheffé multiple-range analysis.

We next determined the activity of transcription factors c-Jun, ATF-2 and Elk-1 in RVLM, which are activated by phosphorylated JNK or p38MAPK [39-41], during experimental brain stem death. Results from ELISA showed that significantly increased ATF-2 activity via phosphorylation at Thr71 in ventrolateral medulla was observed only during the pro-life phase (Figure 3). Similar results were obtained for augmented c-Jun activity via phosphorylation at Ser73, but not for Elk-1 activity as indicated by insignificant phosphorylation at Ser383. On the other hand, the activity of ATF-2, c-Jun or Elk-1 in ventrolateral medulla of aCSF-treatment group was comparable to sham-controls.

### Activation of JNK in RVLM sustains central cardiovascular regulation during experimental brain stem death

**Figure 4:**
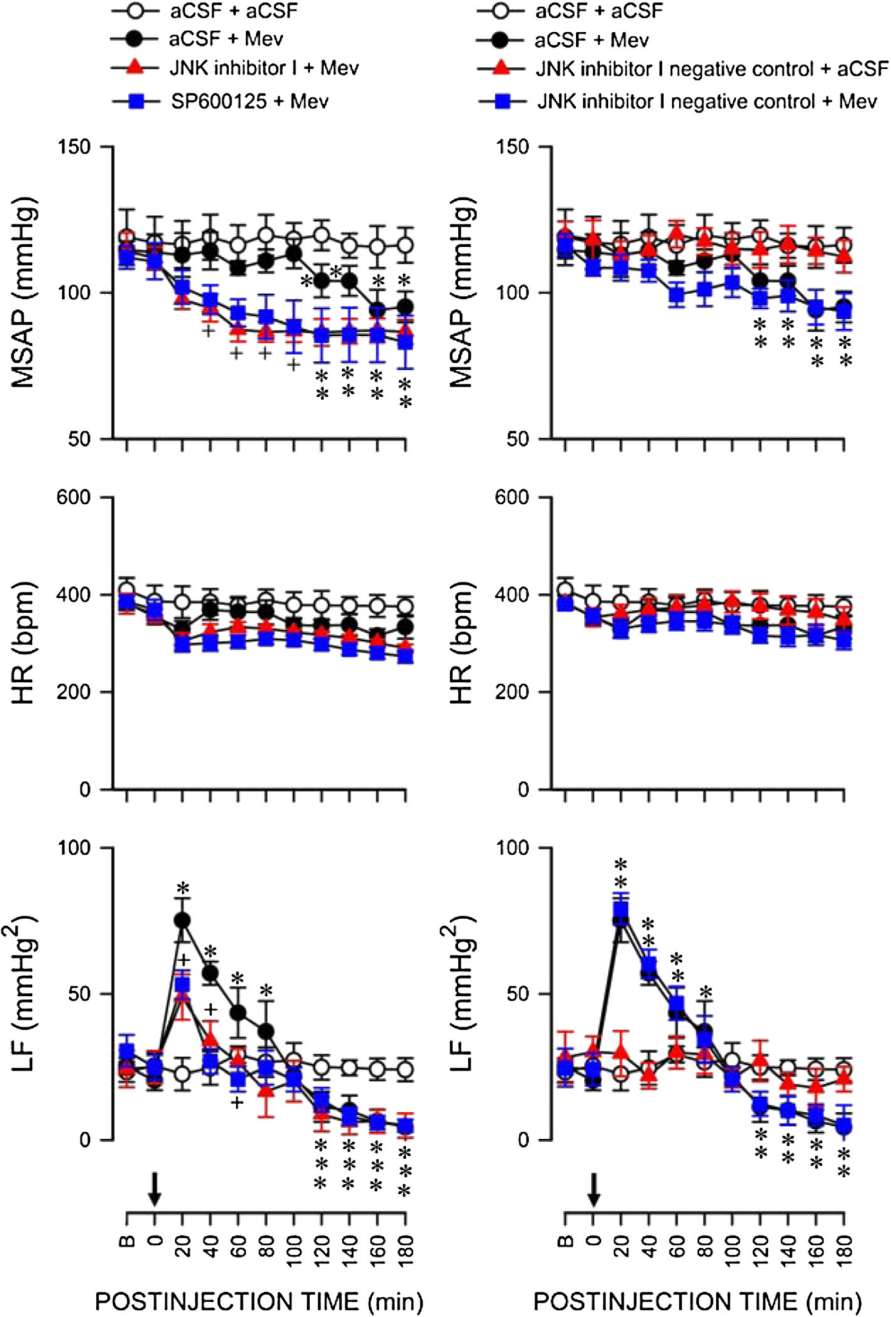
**Activation of JNK in RVLM sustained central cardiovascular regulation associated with experimental brain stem death.** Temporal changes in mean systemic arterial pressure (MSAP), hear rate (HR) or power density of the low-frequency (LF) component of SAP signals in rats that received pretreatment by microinjection bilaterally into RVLM of aCSF (vehicle), JNK inhibitor I (JNK inhibitor), SP600125 (JNK inhibitor) or JNK inhibitor I negative control (negative control for JNK inhibitors), 30 min before local application (at arrow) of aCSF or Mev (10 nmol) to the bilateral RVLM. Values are mean ± SEM, n = 5-7 animals per experimental group. *P < 0.05 versus aCSF+aCSF group, and ^+^*P* < 0.05 versus aCSF+Mev group at corresponding time-points in the post hoc Scheffé multiple-range test.

Based on the stipulation that the magnitude and duration of the LF component of SAP signals during experimental brain stem death reflect the prevalence of the life-and-death signal [6], we next employed pharmacological blockade to evaluate whether a causal relationship exists between activation of JNK in RVLM and central cardiovascular regulation during brain stem death. Pretreatment with microinjection into the bilateral RVLM of JNK inhibitor I (100 pmol), a cell-permeable biological active peptide that binds specifically to JNK to inhibit phosphorylation of the activation domain of JNK and to prevent the activation of the downstream transcription factor c-Jun [35], exacerbated significantly the depressor effect and blunted the augmented power density of the LF component of SAP signals during the pro-life phase (Figure 4), without affecting HR. Similar results were obtained on local application bilaterally into RVLM of SP600125 (5 pmol), a cell-permeable, selective and reversible inhibitor of JNK [36] (Figure 4). Those pretreatments also significantly shortened the pro-life phase to 35–40 min by shifting the prevailing phase of the 180-min observation period toward the pro-death phase (Figure 4). On the other hand, microinjection of JNK inhibitor I negative control (100 pmol) [35] into the bilateral RVLM did not significantly affect the increase in LF power during the pro-life phase nor the depressor effect and decrease in LF power already exhibited during the pro-death phase. Furthermore, pretreatments with aCSF or JNK inhibitor I negative control exerted no significant effects on the minimal cardiovascular responses in the aCSF-control group.

### Activation of p38MAPK in RVLM also sustains central cardiovascular regulation during experimental brain stem death

**Figure 5:**
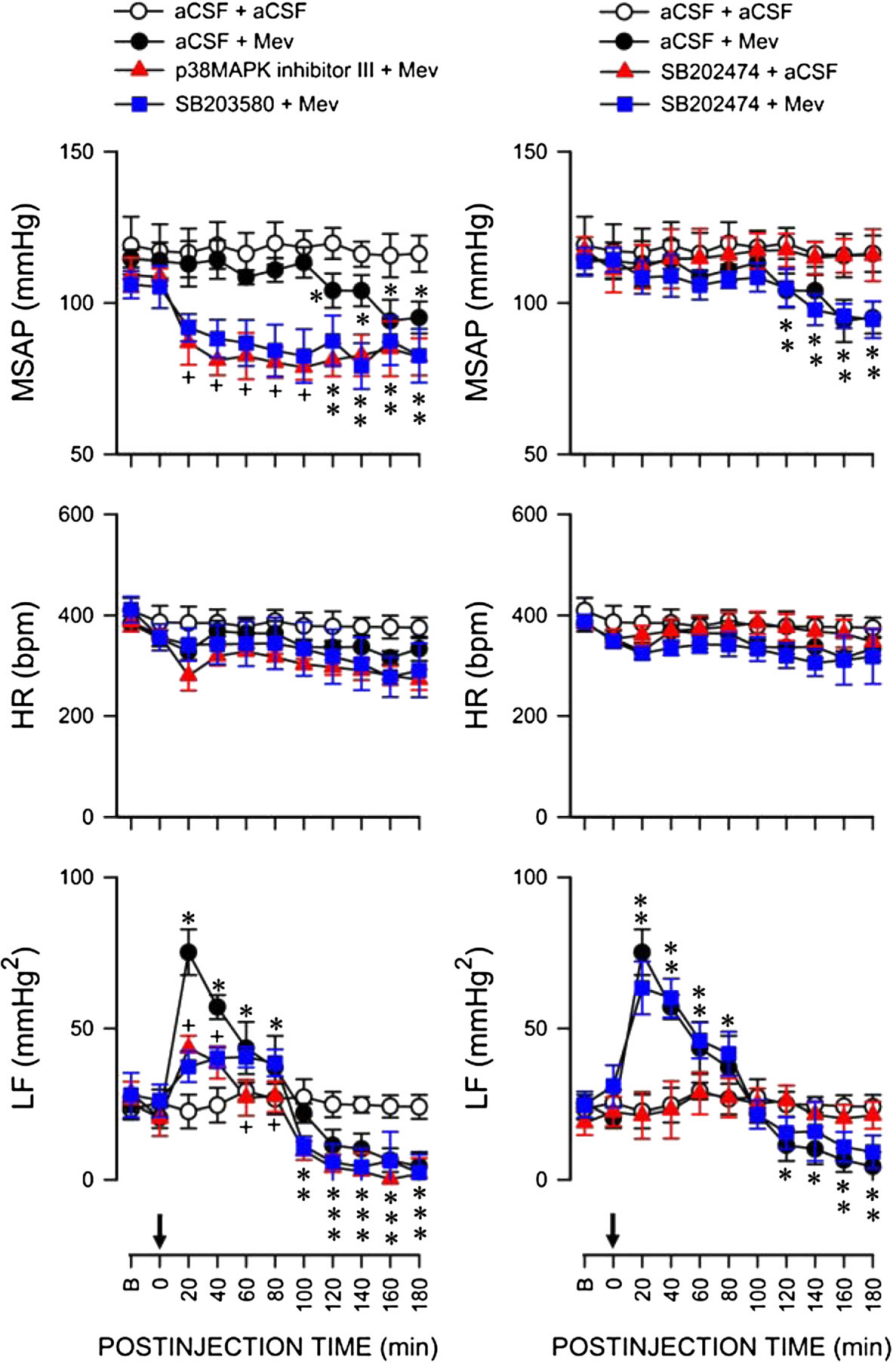
**Activation of p38MAPK in RVLM sustained central cardiovascular regulation associated with experimental brain stem death.** Temporal changes in MSAP, HR or power density of the LF component of SAP signals in rats that received pretreatment by microinjection bilaterally into RVLM of aCSF (vehicle), p38MAPK inhibitor III (p38MAPK inhibitor), SB203580 (p38MAPK inhibitor) or SB202474 (negative control for p38MAPK inhibitors), 30 min before local application (at arrow) of aCSF or Mev (10 nmol) to the bilateral RVLM. Values are mean ± SEM, n = 5-7 animals per experimental group. *P < 0.05 versus aCSF+aCSF group, and ^+^*P* < 0.05 versus aCSF+Mev group at corresponding time-points in the post hoc Scheffé multiple-range test.

We further applied the same experimental scheme to evaluate whether a causal relationship similarly exists between activation of p38MAPK in RVLM and central cardiovascular regulation during experimental brain stem death. Pretreatment with microinjection into the bilateral RVLM of p38MAPK inhibitor III (500 pmol), a potent, selective and ATP competitive p38MAPK inhibitor [37], also exacerbated significantly the depressor effect and blunted the augmented power density of the LF component of SAP signals during the pro-life phase (Figure 5), without affecting HR. Similar results were obtained from SB203580 (2 nmol), a cell-permeable inhibitor of p38MAPK [38] (Figure 5). Those pretreatments also significantly shortened the pro-life phase to 60 min by shifting the prevailing phase of the 180-min observation period toward the pro-death phase (Figure 5). On the other hand, pretreatment with the negative control, SB202474 (2 nmol) was ineffective against the phasic cardiovascular responses in the aCSF-control group or Mev-experimental group.

## Discussion and conclusions

**Figure 6:**
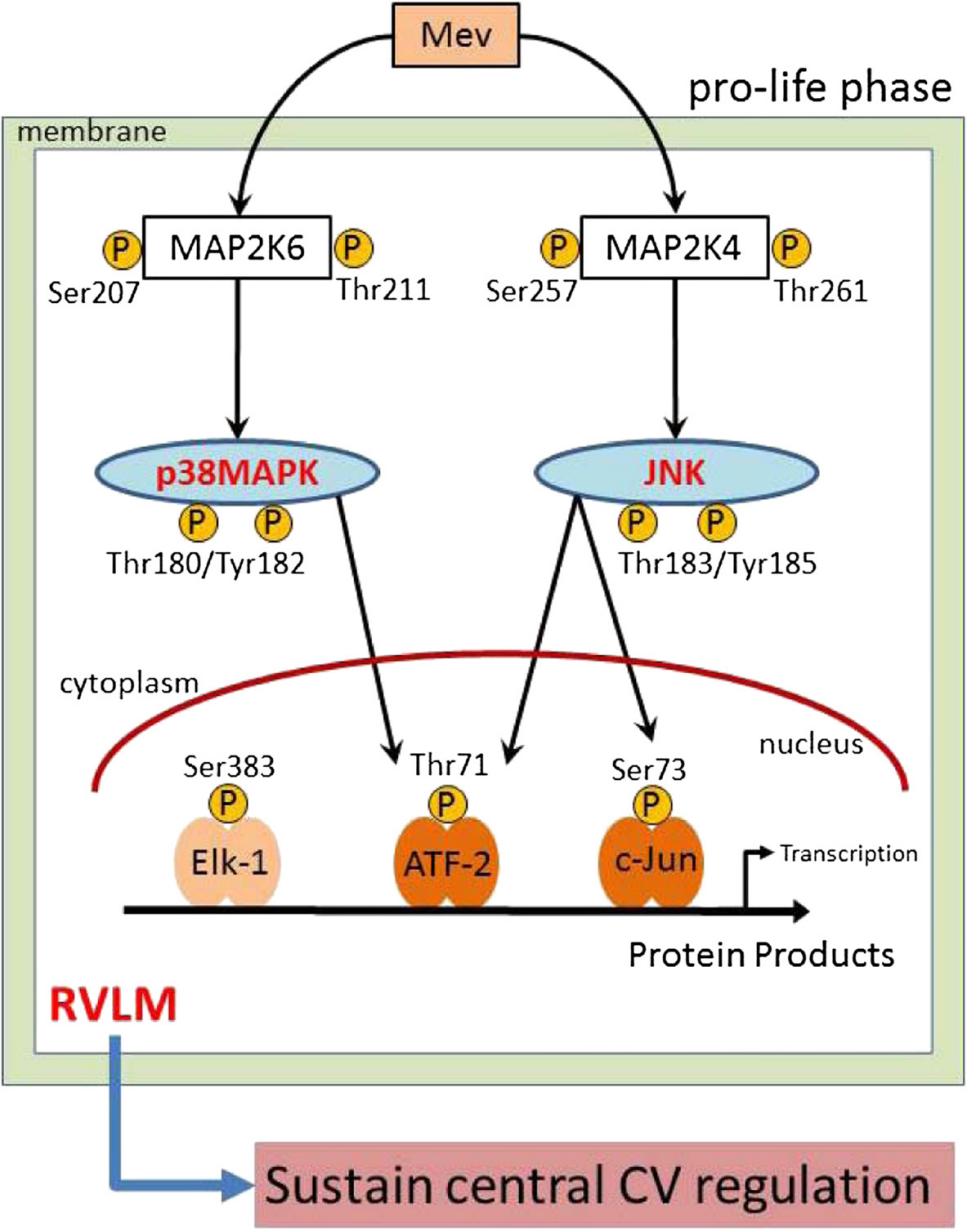
**Schematic summary of the pro-life role of JNK and p38MAPK at RVLM during experimental brain stem death.** Phosphorylation of MAP2K4 or MAP2K6, leading to activation of JNK or p38MAPK, and followed by nuclear activation of transcription factors ATF-2 or c-Jun, but not Elk-1, take place in RVLM preferentially during the pro-life phase of experimental brain stem death. Proteins that are downstream to these signaling cascades in turn sustain central cardiovascular regulation during the progression towards brain stem death. Abbreviation: ATF-2, activating transcriptional factor-2; JNK, c-Jun NH2-terminal kinase; CV, cardiovascular; MAPK, mitogen-activated protein kinase; MAP2K4, MAPK kinase 4; MAP2K6, MAPK kinase 6; Mev, mevinphos; p38MAPK, p38 mitogen-activated protein kinase; RVLM, rostral ventrolateral medulla.

Based on a clinically relevant experimental model [6], the present study provided novel demonstrations that activation of both JNK and p38MAPK in RVLM sustains central cardiovascular regulation during the progression towards brain stem death. We further showed that mechanistically, phosphorylation of MAP2K4 or MAP2K6 is upstream to activation of JNK or p38MAPK during the pro-life phase, with nuclear activation of transcription factors ATF-2 or c-Jun as the downstream signals (Figure 6).

The present study identified a novel pro-life role for MAP2K4/JNK/ATF-2 or c-Jun signaling cascade, rather than Elk-1, in RVLM during experimental brain stem death. JNK is a critical determinant for survival of cardiomyocytes from hypoxia-induced apoptosis [30,42]. Activation of JNK and its downstream transcription factor c-Jun, rather than ERK pathway, also plays a critical role in the survival and proliferation of pulmonary artery endothelial cells induced by epoxyeicosatrienoic acid [43]. Phosphorylation of JNK at Thr183 and Tyr185 by upstream MAP2Ks, MAP2K4 or MAP2K7, is important for the activation of JNK pathway [44,45]. Activation of JNK1/2 by MAP2K4 is responsible for cell survival in primary human umbilical vein endothelial cells mediated by vascular endothelial growth factor receptor-3 [46].

The present study also identified a novel a pro-life role for MAP2K6/p38MAPK/ATF-2 or c-Jun signaling cascade in RVLM during experimental brain stem death. The p38MAPK-dependent signaling cascade mediates critical cellular survival response to stress [47]. Upregulation of p38MAPK plays an important role in survival from cecal ligation and puncture-induced sepsis in mice [48], and inhibits apoptosis or proinflammatory response to lipopolysaccharide in microglial BV-2 cells [26] or in macrophages RAW 264.7 cells [48] or tumor necrosis factor alpha (TNFα) in murine fibrosarcoma L929 cells [49]. On the other hand, a decrease in the expression of phosphorylated p38MAPK is accompanied by cell death in TNFα-treated L929 cells [49]. Constitutive expression of MKK6 (the alternative name for MAP2K6) phosphorylates p38 MAPK and enhances the survival of osteoclasts [50]. Activation of ATF-2 by p38MAPK prevents accumulation of reactive oxygen species and cell death in mouse embryo fibroblast [27].

We demonstrated previously the engagement of hypoxia-inducible factor 1α (HIF-1α)/heme oxygenase 1/heat shock protein 70 (HSP70) signaling pathway induced by hypoxia and tropomyocine receptor kinase B (Trk B)/Ras/Raf signaling pathways activated by brain-derived neurotrophic factor (BDNF) in RVLM during the pro-life phase of experimental brain stem death. Of interest is that a potential role for JNK to serve as a survival factor by phosphorylation of a number of cellular molecules, including c-Jun, AP-1 or Bcl-2, is suggested for myocytes against hypoxia-reoxygenation injury [51]. Decreased JNK phosphorylation induced by inhibition of Ras or Raf mediates cell apoptosis [52]; and inhibition of Ras and p38MAPK reduces BDNF-induced survival of ganglion neurons [53]. Activation of the p38MAPK pathway is also an early response to hypoxia for cell survival because p38MAPK inhibition abolishes cell survival from hypoxia in rat neonatal cardiac myocytes [54] or LNCaP cells [55] and phosphorylation of p38MAPK induced by hypoxia-preconditioning mediates the protection of cardiomyocyte from ischemic injury [56]. It follows that JNK or p38MAPK may participate in the pro-life phase of experimental brain stem death as a consequence of hypoxia or BDNF activation in RVLM. Further studies are required to delineate these implied signaling cascades.

The transcription factor c-Jun is one of the most consistent markers for neuronal fate and is determined by a transcriptional network comprising c-Jun, ATF-2 and JNKs [57]. Overexpression of c-Jun in rat pheochromocytoma PC12 cells renders them to be more resistant to apoptosis induced by okadaic acid [58] or serum-deprivation [59]. High levels of c-Jun mRNA and proteins even function as a neuronal survival or neurite outgrowth signal for PC12 cell [58]. Mechanistically, it is most likely that ATF-2 or c-Jun in RVLM participates in the pro-life process by regulating its target proteins transcriptionally. Some of the known candidate proteins include HIF-1α [60,61], HSP70 [62,63], anti-apoptotic Bcl-XL [64] and neuronal nitric oxide synthase [65]. In addition to transcriptional regulation, c-Jun also mediates posttranscriptional modification on HIF-1α by protecting it from proteasomal degradation [66]. Interestingly, all these proteins have been found to play a pro-life role in RVLM in our experimental model of brain stem death [16,67-71]. Fischer et al., [72] reported that marked increases in JNK and p38MAPK activity, coincident with an increase in phosphorylation of c-Jun and ATF-2, can be detected as early as 15–30 min after rapid changes in hemodynamic load in Wistar rats. This time-course befits an active role for c-Jun and ATF-2 in RVLM during the pro-life phase of experimental brain stem death.

In conclusion, the present study demonstrated that the MAP2K4/JNK or MAP2K6/p38MAPK signaling cascade in RVLM plays a pro-life role during experimental brain stem death by sustaining the central cardiovascular regulatory machinery via activating the transcription factors ATF-2 or c-Jun. This information provides further insights into the cellular mechanisms of brain stem death, and offers new targets for the development of therapeutic interventions against this fatal phenomenon.

## Competing interests

The author declares that she has no competing financial interests.

## Authors’ contributions

AYWC conceived the study, participated in experimental design, and drafted and revised the manuscript.
